# Basal Cancer Cell Survival Involves JNK2 Suppression of a Novel JNK1/c-Jun/Bcl-3 Apoptotic Network

**DOI:** 10.1371/journal.pone.0007305

**Published:** 2009-10-06

**Authors:** Shafiq Uddin Ahmed, Jo Milner

**Affiliations:** YCR P53 Research Unit, Department of Biology, University of York, York, United Kingdom; University of Texas MD Anderson Cancer Center, United States of America

## Abstract

**Background:**

The regulation of apoptosis under basal (non-stress) conditions is crucial for normal mammalian development and also for normal cellular turnover in different tissues throughout life. Deficient regulation of basal apoptosis, or its perturbation, can result in impaired development and/or disease states including cancer. In contrast to stress-induced apoptosis the regulation of apoptosis under basal conditions is poorly understood. To address this issue we have compared basal- and stress-induced apoptosis in human epithelial cells of normal and cancerous origins. For this purpose we focussed our study on the opposing pro-apoptotic JNK/anti-apoptotic NFκB pathways.

**Methodology/Principal Findings:**

Combinatorial RNAi plus gene knockout were employed to access and map basal regulatory pathways of apoptosis. Follow-on, in-depth analyses included exogenous expression of phosphorylation mutants and chromatin immunoprecipitation. We demonstrate that basal apoptosis is constitutively suppressed by JNK2 in a range of human cancer cell lines. This effect was not observed in non-cancer cells. Silencing JNK2 by RNAi resulted in JNK1-dependent apoptosis of cancer cells via up-regulation of the AP-1 factor c-Jun. Unexpectedly we discovered that JNK1 and c-Jun promote basal apoptosis in the absence of “activating phosphorylations” typically induced by stress. Hypo-phosphorylated c-Jun accumulated to high levels following JNK2 silencing, auto-regulated its own expression and suppressed expression of Bcl-3, an unusual IκB protein and regulator of NFκB. Basal apoptosis was mediated by components of the TNFα response pathway but was mechanistically distinct from TNFα-induced apoptosis.

**Conclusions/Significance:**

Our results demonstrate that mechanistically distinct pathways operate to regulate apoptosis in mammalian cells under basal (physiological) versus stress-induced conditions. We also describe a novel apoptotic network which governs the basal survival of cancer cells. Such information is crucial for understanding normal cellular turnover during mammalian development and subsequently throughout life. This information also opens new avenues for therapeutic intervention in human proliferative disease states including cancer.

## Introduction

The c-Jun N-terminal kinases (JNKs) are members of the mitogen-activated protein kinase family (MAP kinases, MAPKs) and are activated in response to cellular stress [1], [2]. In response to stress JNK1 and JNK2 are activated via dual phosphorylation of T183 and Y185 by MAPK kinases (MAPKK), specifically MKK4 and MKK7 [3], [4]. MAPKs and MAPKKs form part of a signal transduction super-family that includes the extracellular signal-regulated kinases (ERKs) and p38 MAPK. The functions of JNK1 and JNK2 are determined by cell type and by the nature of the stress responsible for their activation. Initial studies identified JNKs by their ability to phosphorylate the N-terminus of c-Jun, a member of the activating protein 1 (AP-1) transcription factor family [5]. Subsequently JNKs were shown to phosphorylate and regulate the activity of other AP-1 proteins as well as additional proteins involved in cell proliferation and apoptosis including p53, c-Myc, Bcl-2 and Bim [2], [6].

AP-1 factors are a group of structurally and functionally related members of the Jun protein family (c-Jun, JunB and JunD) and the Fos protein family (c-Fos, FosB, Fra-1 and Fra-1) [7]. Dimerisation within these family members forms an AP-1 transcription factor and the relative abundance of individual AP-1 subunits and dimer composition are important factors determining cell fate. The repertoire of AP-1 dimer composition allows tailoring of an AP-1-mediated response by individual cell types to a given stimulus. Post-translational modification and protein turnover are two mechanisms for regulating AP-1 activity. For example, in response to stress the transactivation potential of c-Jun is activated by JNK-mediated N-terminal phosphorylation [8], [9], whereas the stability of c-Jun protein is down-regulated via GSK-3-mediated C-terminal phosphorylation which targets c-Jun for ubiquitinylation and degradation via the E3 ligase Fbw7 [10].

An important activator of the JNK apoptotic pathway is tumour necrosis factor α (TNFα), a pro-inflammatory cytokine that governs cell survival via promoting either cell proliferation or apoptosis [11]. TNFα engages with its trimeric receptor, TNFR1, at the external surface of the cell membrane. TNFα/TNFR1 interaction results in formation of an intracellular heterogeneous protein complex (complex 1) at the cytoplasmic tail of TNFR1. In its turn this complex leads to activation of JNK and also of IκB kinase (IKK). Elegant studies *in vitro* and *in vivo* have demonstrated that, in hepatocytes exposed to TNFα, activated JNK1 phosphorylates and activates the E3 ubiquitin ligase ITCH [1], [12]. Activated ITCH induces ubiquitinylation and degradation of cFLIP (cellular FLICE-inhibitory protein) which otherwise specifically inhibits activation of pro-caspase 8 (also called FLICE). Caspase 8 activation results in apoptosis [1], [10]. This pro-apoptotic pathway is counter-balanced by NFκB which up-regulates c-FLIP expression and thus inhibits pro-caspase 8 activation and apoptosis [13].

NFκB is a transcription factor composed of homo- or heterodimers of members of the Rel family of proteins, including p65 (RelA), RelB, c-Rel, p50 and p52 (reviewed in [14]. Transcription activation domains are absent in p50 and p52 which thus need to form heterodimers in order to transactivate target genes. The principal assembly is a heterodimer of p65–p50 but different dimer compositions are also formed. NFκB is subject to regulation by protein-protein interactions with IκB proteins. IκBα and IκBβ preferentially target p65–p50 heterodimers. Phosphorylation of IκB by IκB kinases triggers IκB degradation with release of dimeric NFκB which translocates into the nucleus and regulates gene expression. Bcl-3 is an unusual IκB which preferentially binds p50–p50 and p52–p52 NFκB homodimers [15], [16]. Bcl-3 is further distinguished from other IκB proteins in that it can localise to the nucleus, it is not degraded upon activation of NFκB and it possesses seven, rather than six, ankyrin repeat domains. Nuclear localisation of Bcl-3 is compatible with its ability to form a ternary complex with DNA-bound p50:p50 homodimers of NFκB, and also to assist uptake of p50 into the nucleus (see [17] and references therein). Bcl-3 was first described in B-cell chronic lymphocytic leukaemia [18] and is regarded as a putative oncoprotein.

In addition to their roles in the stress response JNK1 and JNK2 also function under basal, non-stress conditions as evidenced by tissue-specific abnormalities observed in JNK1−/− and JNK2−/− mice. For example, JNK1−/− mice exhibit decreased T-cell differentiation and defective immunity [19]. Importantly JNK1−/− mice also develop spontaneous intestinal tumours, indicating that JNK1 functions as a tumour suppressor for this tissue type [20]. However, the mechanisms of JNK1 and JNK2 functioning under basal, physiological conditions are poorly understood.

To investigate the individual roles of JNK1 and JNK2 in human epithelial cells under basal conditions we employed a combination of (i) RNAi induced in the absence of applied stress, (ii) gene knock-out cell lines, and (iii) exogenous gene expression. In a range of human cell lines we discovered that JNK1 is actively pro-apoptotic but is constitutively inhibited by the presence of JNK2 in cancer cells. This effect was not observed in non-cancer cells. Co-silencing experiments revealed that these basal, opposing pro- and anti-apoptotic functions of JNK1 and JNK2 are largely independent of up-stream kinases MKK4 and MKK7. This was consistent with lack of discernable change in JNK1 phosphorylation despite JNK1-dependent apoptosis following depletion of JNK2. Further experiments revealed that the basal JNK1 pro-apoptotic pathway involves c-Jun plus components of the TNFα-responsive MAP kinase pathway coupled with the NFκB pathway via c-Jun-mediated down-regulation of Bcl-3. Thus the basal regulation of cancer cell survival appears to be dependent upon the opposing functions of JNK2/Bcl-3 (pro-survival) and JNK1/c-Jun (pro-apoptotic) and to be under constitutive control by JNK2.

## Results

### Selective knock-down of JNK2 enhances JNK1 expression

**Figure 1:**
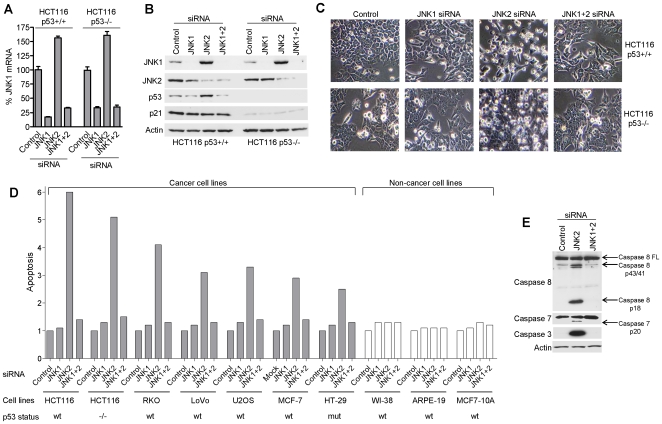
JNK2 constitutively suppresses JNK1-mediated apoptosis in a range of human cancer cell lines but not in non-cancer cells. (A) JNK1 mRNA levels, and (B) JNK1, JNK2, and also p53 and p21 protein levels determined 48 h after transfection with JNK1- and JNK2-siRNAs as indicated (Methods). (C) Phase contrast images of HCT116 cells 48 h after transfection with JNK1- and JNK2-siRNAs. (D) Apoptosis determined by annexin V labelling of siRNA-treated cells relative to untreated controls in a range of human cell lines (Methods. Note: apoptosis results are representative of at least three repeat experiments unless error bars are included for repeat analyses of a single experiment). (E) Caspase 8 and its cleavage products, activated caspase 7 and effector caspase 3 following RNAi-mediated silencing of JNK1 and JNK2 as indicated. Actin  =  loading controls. HCT116 p53+/+ and HCT116 p53−/− are isogenic clones of human HCT116 colorectal cancer cells (Methods).

For RNAi we employed synthetic siRNAs under conditions previously demonstrated to give efficient knock-down of target mRNAs without activation of the p53 cellular stress response [21], [22]. RNAi controls included BCR-ABL siRNA, which has no mRNA target in epithelial cells and therefore serves as a non-functional siRNA control, and lamin A/C siRNA, which targets lamin A/C mRNA and serves as a functional siRNA control (Methods). Selective knock-down of JNK1 and JNK2 mRNA was reproduced with two different siRNAs for each target (Methods and Fig. S1). The efficiency of mRNA knock-down was determined by quantitative PCR and was similar for both JNK1 and JNK2 (∼75% reduction; see Fig. 1A for JNK1 mRNA levels). RNAi-induced silencing of lamin A/C or of JNK1 did not induce any change in p53 or p21^WAF1^, a p53-dependent target gene (Fig. 1B for JNK1 siRNA and Fig. S2 for lamin A/C siRNA), thus confirming that the conditions of RNAi *per se* do not initiate a p53 stress response. However JNK2 silencing resulted in a ∼3 fold increase in p53 protein (Fig. 1B) suggesting that JNK2 directly or indirectly regulates the turnover of p53 protein. This is consistent with the report that JNKs can regulate the basal turnover of p53 [23]. Interestingly, co-silencing of JNK1 with JNK2 abrogated the rise in p53 (Fig. 1B) implying that JNK1 and JNK2 exert co-ordinated effects upon the turnover of p53.

Unexpectedly, in HCT116 colorectal cancer cells JNK2 knock-down consistently resulted in a ∼50% increase in JNK1 mRNA levels and a ∼3 fold increase in JNK1 protein levels (Fig. 1A and 1B). Similar results for JNK1 mRNA were obtained in isogenic HCT116 p53^+/+^ and HCT116 p53^−/−^ cells (Fig. 1A and 1B), indicating that the effect is p53-independent. Thus, in HCT116 cells, the presence of JNK2 directly or indirectly suppresses basal JNK1 expression levels.

### JNK2 knock-down induces apoptosis

Knock-down of JNK2 resulted in apoptosis in a range of cancer cell lines including HCT116 cells (Fig. 1C and 1D). Apoptosis was determined by annexin V staining (Methods) and involved pro-caspase 8 activation and effector caspases 3 and 7 (Fig. 1E). This apoptotic effect of JNK2 depletion was independent of p53 as evidenced by HCT116 p53−/− cells (Fig. 1C and 1D). In non-cancer cells JNK2 silencing did not induce apoptosis (ARPE19 and MCF10A epithelial cells, and WI-38 fibroblasts) despite efficient JNK2 mRNA knock-down (Fig. 1D and data not shown). In contrast to JNK2 silencing, the silencing of JNK1 did not affect viability in any of the cell lines tested (Fig. 1C and 1D). Overall these results indicate that JNK2, but not JNK1, is essential for basal viability in a number of human cancer cell lines including MCF7 breast cancer epithelial cells, but is dispensable for basal viability of non-cancer cells including MCF10A breast epithelial cells.

### Apoptosis is JNK1-dependent

We next co-silenced JNK1 with JNK2 in order to investigate their functional linkage in the regulation of cancer cell survival. In all cases where JNK2 silencing induced apoptosis we found that co-silencing JNK1 with JNK2 rescued JNK2-depleted cells from apoptosis (Fig. 1D). Thus we conclude that JNK1 and JNK2 counter-balance each other in the maintenance of cancer cell viability and that JNK2 constitutively suppresses JNK1-mediated apoptosis under basal conditions.

### JNK1-dependent apoptosis is independent of enhanced JNK1 S63/73 phosphorylation

The above results indicate that JNK1 is primed to induce apoptosis in human epithelial cancer cells but is constitutively suppressed by JNK2. Both JNK1 and JNK2 are downstream mediators of the MAP kinase apoptotic pathway and are activated in response to stress by MKK4 and/or MKK7 phosphorylation at T183/Y185 (Introduction). We next compared JNK1 and JNK2 phosphorylation under basal (RNAi) and stress-induced (UV or TNFα) conditions.

**Figure 2:**
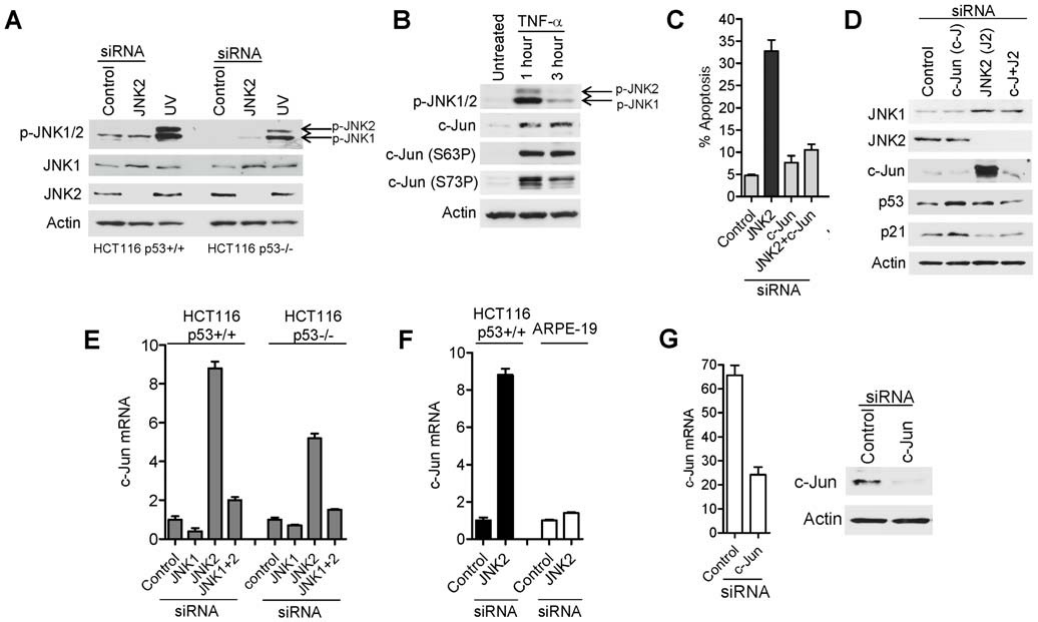
JNK1 and c-Jun, required for basal apoptosis, remain hypo-phosphorylated following JNK2 silencing whereas both are hyper-phosphorylated in response to stress. (A) Comparison of JNK1/JNK2 phosphorylation status in control, JNK2-silenced and following UV-irradiation (HCT116 cells; Methods). p-JNK1  =  phosphorylated JNK1, pJNK2  =  phosphorylated JNK2 (arrowed). (B) Effects of TNFα on phosphorylation status of JNK1, JNK2 and c-Jun. (C) Co-silencing c-Jun with JNK2 rescues HCT116 cells from apoptosis (annexin V labelling; Methods). (D) JNK1, JNK2 and c-Jun protein levels 48 h post RNAi-mediated silencing of JNK2 and c-Jun. P53 and p21 protein levels are also shown. (E) c-Jun mRNA levels after JNK1 and JNK2 silencing. (F) Fold increase in c-Jun mRNA levels in HCT116 cells compared with ARPE19 cells 48 h after JNK2 silencing. (G) Knockdown of c-Jun mRNA and protein levels in HCT116 p53+/+ cells following c-Jun siRNA treatment.

As expected UV irradiation induced strong phosphorylation at residues T183/Y185 of both JNK1 and JNK2 proteins in HCT116 cells (Fig. 2A). Similarly, treatment of HCT116 cells with TNFα also induced JNK1 and JNK2 phosphorylation at T183/Y185 (Fig. 2B, upper panel). In this latter instance JNK1 phosphorylation predominated over JNK2 phosphorylation. These results clearly demonstrate phosphorylation of JNK1 and JNK2 in response to genotoxic stress (UV) and to receptor-mediated stress (TNFα) in HCT116 cells. Both UV-irradiation and TNFα induced apoptosis (data not shown).

Under basal conditions, when JNK1 was actively pro-apoptotic following JNK2 silencing, there was no discernable increase in JNK1 T183/Y185 phosphorylation (Fig. 2A, compare control lane with JNK2 siRNA lane). This is in striking contrast to stress-induced conditions (Fig. 2A; compare JNK2 siRNA lane with UV lane). Thus we infer that JNK1 is able to promote apoptosis in the absence of ‘activating’ T183/Y185 phosphorylations that are characteristically induced in response to stress. Co-silencing up-stream kinases MKK4 or MKK 7 with JNK2 only partially rescued JNK1-dependent apoptosis (Fig. S3), further substantiating the ability of JNK1 to promote apoptosis in the absence of enhanced T183/Y185 phosphorylations.

We conclude that, under basal conditions, JNK1 can actively promote apoptosis without undergoing enhanced T183/Y185 phosphorylation characteristic of the cellular stress response. This basal pro-apoptotic function of JNK1 is evident in human epithelial cancer cells (but not in non-cancer cells) and is constitutively inhibited by endogenous JNK2. To investigate the basal apoptotic pathway under JNK1 control we next carried out a series of co-silencing experiments aimed at identifying essential pro-apoptotic mediators linked with the JNK1-dependent apoptosis following JNK2 silencing.

### c-Jun is an essential mediator of basal apoptosis and is subject to opposing effects of JNK1 and JNK2

To ask if c-Jun is required for apoptosis under basal conditions we co-silenced c-Jun with JNK2 (Fig. 2; the efficiency of c-Jun mRNA knockdown is shown in Fig. 2G). The results clearly demonstrate that c-Jun co-silencing rescues JNK2-silenced HCT116 cells from apoptosis thus identifying c-Jun as an essential mediator of apoptosis subject to JNK2 suppression under basal conditions (Fig. 2C).

**Figure 3:**
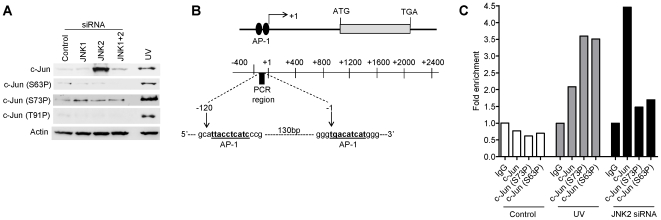
Hypo-phosphorylated c-Jun protein accumulates to high levels following JNK2 silencing, this effect is dependent upon JNK1 and correlates with c-Jun chromatin binding at the c-Jun promoter in HCT116 cells. (A) Total c-Jun protein and phosphorylations at S63, S73 and T91 following either UV-irradiation (right hand lane) or JNK1 and JNK2 RNAi-induced silencing. (B) Schematic showing *c-Jun* and the amplicon used for detecting c-Jun on the promoter. (C) Total and phosphorylated forms of c-Jun bound at the c-Jun promoter detected by chromatin immunoprecipitation (ChIP) with c-Jun and c-Jun phospho-specific antibodies. Histogram shows fold-enrichment relative to IgG controls (Methods).

The c-Jun protein accumulated to high levels following JNK2 silencing in HCT116 cells (Fig. 2D; increased c-Jun was also observed in RKO and Lovo cells following JNK2 silencing, not shown) paralleled by an increase in c-Jun mRNA (Fig. 2E). Interestingly, this effect was abolished when JNK1 was co-silenced with JNK2 (Fig. 3A for c-Jun protein and Fig. 2E for c-Jun mRNA) indicating that up-regulation of c-Jun is dependent upon the continued presence of JNK1. JNK1 silencing alone caused a decrease in c-Jun mRNA and protein (Fig. 2E and Fig. S4). These combined observations indicate that JNK1 and JNK2 exert opposing effects upon c-Jun expression and that JNK1 is required for maintenance of basal c-Jun mRNA and protein expression levels, whilst JNK2 constitutively suppresses c-Jun mRNA and protein expression levels. Similar results were observed in HCT116 p53^+/+^ and HCT116 p53^−/−^ cells (see for example Fig. 2E). Interestingly increased c-Jun mRNA levels were not observed in JNK2-silenced ARPE19 cells (Fig. 2F) consistent with lack of apoptosis in these cells (Fig. 1D).

Our results identify opposing effects of JNK1 and JNK2 on c-Jun. In this respect they differ from observations obtained using a ‘chemical genetics’ approach in which the catalytic ATP-binding pocket of JNK2 was expanded by mutation [2]. The conclusion from this latter approach was that JNK1 and JNK2 both positively regulate c-Jun expression. However, it is possible that expansion of the ATP-binding pocket of JNK2 may perturb the spatial molecular organisation of the adjacent β-strand-like region of JNK2 protein. Since this β-strand-like domain is important for JNK2 protein-protein interactions we suggest that localised perturbation at this site may compromise the binding characteristics of JNK2 protein for its target substrates. This might affect the functioning of mutant JNK2 in ways additional to the predicted effects on molecular access into the expanded ATP binding pocket of JNK2.

Another study comparing murine fibroblasts derived from JNK1−/− and JNK2−/− mice indicated that JNK1 and JNK2 can function as opposing regulators of c-Jun [3]. Our own results, under basal conditions, are consistent with this report and show that in HCT116 cells (i) JNK2 silencing results in up-regulation of c-Jun mRNA accompanied marked accumulation of c-Jun protein, and (ii) up-regulation of c-Jun under these conditions is dependent upon JNK1.

### Up-regulation of JNK1 in JNK2-depleted cells is independent of c-Jun

Given that basal up-regulation of c-Jun (in JNK2-depleted cells) requires JNK1 (see above) we wondered if c-Jun, in its turn, is required for the up-regulation of JNK1 protein following JNK2 silencing (Fig. 1B). However the increase in JNK1 consistently observed in JNK2-silenced cells was unaffected by co-silencing c-Jun with JNK2 (Fig. 2D; upper panel). This indicates that enhanced mRNA and protein expression of JNK1 following JNK2 depletion is independent of c-Jun.

### Hypo-phosphorylated c-Jun binds the c-Jun promoter

Stress-induced S63/S73 phosphorylation of c-Jun by JNK kinases releases c-Jun from an inhibitory complex thus enabling c-Jun to bind and transactivate target promoters [24]. Stress-induced phosphorylation of c-Jun in HCT116 cells treated with UV-irradiation is shown in Fig. 3A. However, although we observed high accumulation of c-Jun protein following JNK2 silencing (Fig. 3A; JNK2 siRNA lane) this was independent of increased N-terminal phosphorylation at S63, S73 or at S91 (Fig. 3A).

JNK2 silencing induces a marked increase in c-Jun mRNA levels (see above, Fig. 2E). To ask if increased c-Jun transcripts were attributable, at least in part, to a positive auto-regulatory feed-back loop we performed chromatin immunoprecipitation (ChIP) for c-Jun/c-Jun promoter complexes (Fig. 3B and 3C). In untreated HCT116 cells c-Jun was undetectable at the c-Jun promoter (Fig. 3C, controls). Following UV-irradiation c-Jun was phosphorylated at S63 and S73 (Fig. 3A) and the phosphorylated protein was bound at the c-Jun promoter as indicated by ChIP analysis using anti-phospho-T63/73 c-Jun antibodies (Fig. 3C). In JNK2-depleted cells, under basal conditions, no change in c-Jun T63/73 phosphorylation was evident relative to the controls (Fig. 3A). Nonetheless c-Jun in these cells was bound at the c-Jun promoter (Fig. 3C). Lack of phosphorylation of the chromatin-bound c-Jun was confirmed by lack of reactivity with anti-phospho-T63/73 c-Jun antibodies (Fig. 3C; compare samples from UV-irradiated cells with JNK2-silenced cells). Thus we conclude that S63/73 hypo-phosphorylated c-Jun binds to its own promoter following JNK2 depletion and is likely to contribute to the high c-Jun mRNA expression levels (∼9-fold increase over control, see Fig. 2E) induced under these basal conditions.

### c-Jun protein accumulation correlates with loss of phosphorylation at S243

**Figure 4:**
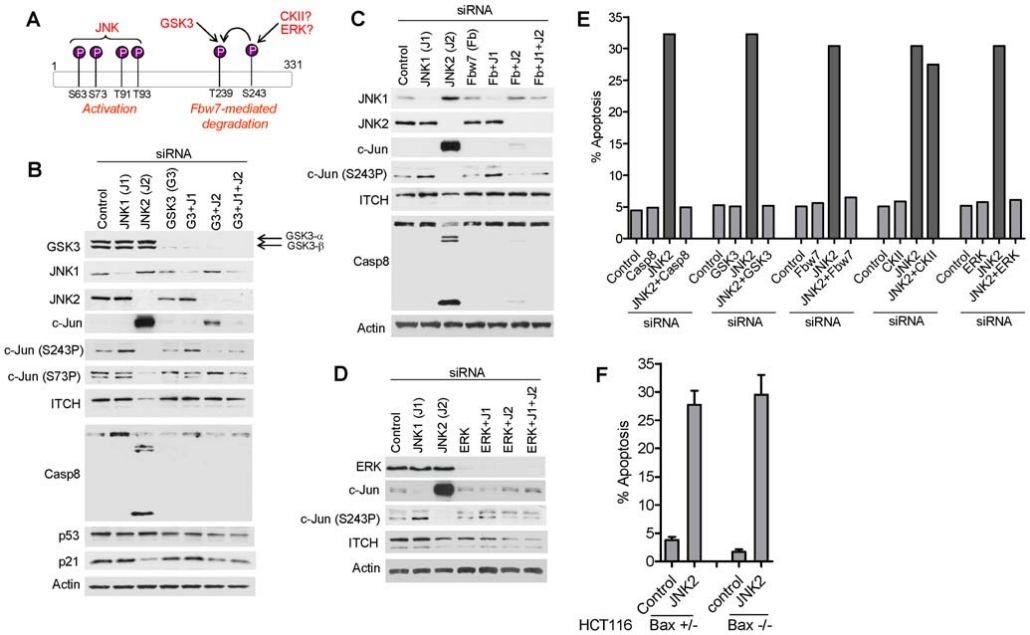
GSK3, ERK and Fbw7 are essential basal pro-apoptotic mediators following JNK2 silencing but are without apparent effect upon their stress-associated substrate c-Jun. (A) Schematic showing sites of phosphorylation of c-Jun by JNK, GSK-3 and ERK, and their respective functional consequences. Note that S243P is a pre-requisite of T239 phosphorylation (indicated by curved arrow). (B) Immunoblots showing effects of JNK1, JNK2 and GSK3 silencing, alone and in combinations, on a panel of HCT116 cellular proteins. (C) and (D) Immunoblots following combinatorial silencing of JNK1 and JNK2 with Fbw7 and ERK. (E) Essential pro-apoptotic intermediates determined by co-silencing with JNK2. (F) Apoptosis following JNK2 silencing under basal conditions is independent of Bax as evidenced by Bax+/− and Bax−/− HCT116 isogenic clones.

It is possible that c-Jun is maintained at low levels in HCT116 cells, lower than observed for non-cancer ARPE19 cells (data not shown), via continual c-Jun protein degradation. Degradation of c-Jun involves C-terminal phosphorylation at S243 which primes c-Jun for GSK3-mediated phosphorylation at T239. This generates an attachment site for the Fbw7 E3 ubiquitin ligase, resulting in polyubiquitinylation and c-Jun degradation [10]. In our present study we noted that JNK1 silencing caused a slight but reproducible increase in c-Jun S243P (see Fig. 4B–4D; control lane cp JNK1 siRNA lane S243P immunoblots). Conversely JNK2 silencing reproducibly caused reduced levels of c-Jun 243P, sometimes undetectable despite massive accumulation of total protein (Fig. 4B–4D; control lane cp JNK2 siRNA for S243P immunoblots). Thus JNK1 and JNK2 exert opposing effects upon c-Jun phosphorylation at S243, a modification linked with de-stabilisation of c-Jun protein.

### GSK3, ERK, Fbw7 and ITCH are essential mediators of basal JNK1-dependent apoptosis

In a further series of co-silencing experiments we demonstrate that kinases GSK3, ERK, and the ubiquitin 3 ligase Fbw7 are also essential mediators of JNK1-dependent apoptosis in JNK2-depleted cells (Fig. 4E). However none of these components appeared to impinge upon S243 phosphorylation or accumulation of the c-Jun protein (Fig. 4B, 4C and 4D; lanes GSK3, Fbw7 and ERK respectively). This was unexpected since, in response to stress, all three have been linked with c-Jun degradation via S239 and S243 phosphorylation (ERK and GSK3 respectively) and ubiquitin-mediated degradation (Fbw7) [10], [25], [26]. It seems that, under basal conditions, GSK3, ERK and Fbw7 function as essential mediators of apoptosis via substrates other than c-Jun.

### Bax and CKII are dispensable for the basal JNK1/JNK2 apoptotic pathway

Following stress Bax is a pro-apoptotic mediator of JNK-induced apoptosis [27], [28]. To ask if Bax is also required for the basal JNK1/JNK2-regulated apoptotic pathway we used isogenic Bax+/− and Bax−/− HCT116 cells [2]. JNK2 silencing induced apoptosis in both HCT116 Bax^+/−^ and HCT116 Bax^−/−^ cells (Fig. 4F) indicating that Bax is not required for apoptosis under these basal conditions. Co-silencing CKII, a putative kinase for c-Jun [3], also failed to rescue JNK2-depleted HCT116 cells from apoptosis (Fig. 4E). Thus both Bax and CKII appear dispensable for the basal JNK1/JNK2 apoptotic pathway.

### Interaction between c-Jun and ITCH

**Figure 5:**
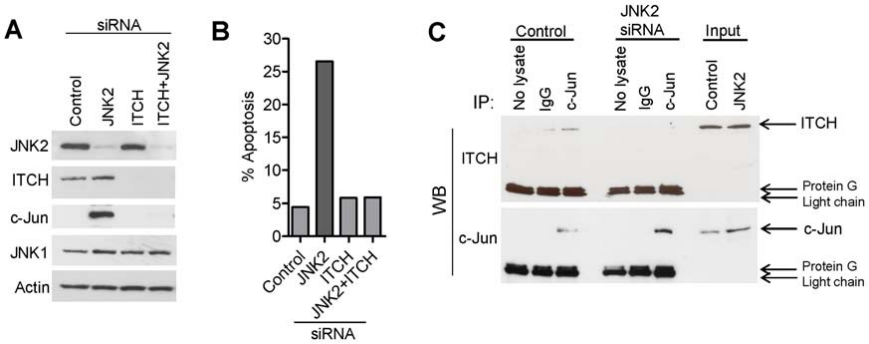
c-Jun is complexed with ITCH under basal condition and released following JNK2 silencing. (A) and (B) Protein and apoptotic levels observed following ITCH and JNK2 silencing in HCT116 cells. (C) Complexes of endogenous ITCH-c-Jun proteins detected prior to JNK2 silencing are lost following JNK2 depletion.

The pro-apoptotic pathway induced by TNFα culminates in phosphorylated JNK1-dependent activation of ITCH, degradation of c-FLIP and caspase 8 activation (see Introduction). Here we show that ITCH is also an essential pro-apoptotic mediator governed by JNK1/JNK2 under basal conditions and that co-silencing ITCH with JNK2 rescues cells from apoptosis (Fig. 5A and 5B).

To further investigate the pro-apoptotic role of ITCH under basal conditions we performed immunoprecipitation/immunoblot analysis for endogenous complexes of ITCH-c-Jun before and after RNAi-mediated silencing of JNK2 (Fig. 5C). Significantly, ITCH-c-Jun complexes were detectable under control conditions but these complexes were lost following JNK2 silencing (Fig. 5C).

### Apoptosis proceeds via c-FLIP and caspase 8

**Figure 6:**
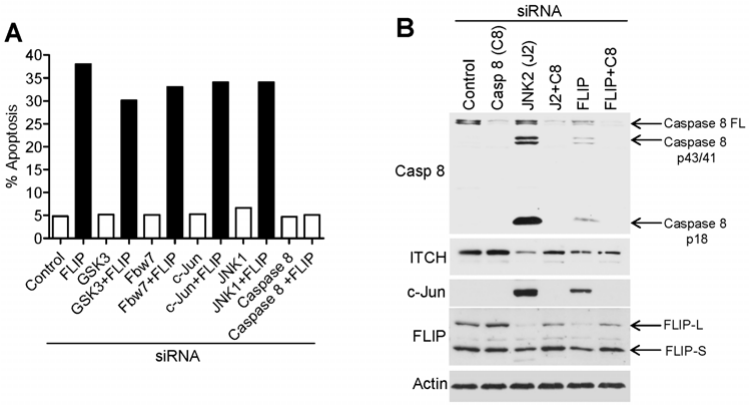
c-FLIP is an essential mediator of basal apoptosis in human cancer cells and functions downstream of GSK3, Fbw7, c-Jun and JNK1. (A) Levels of apoptosis observed following co-silencing of c-FLIP with pro-apoptotic mediators. (B) Protein levels showing pro-caspase 8 cleavage and c-Jun accumulation following c-FLIP depletion by RNAi.

**Figure 7:**
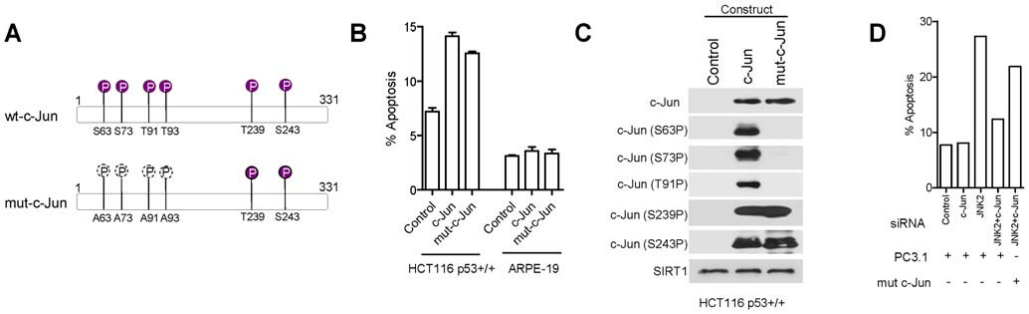
c-Jun overexpression induces apoptosis in HCT116 p53+/+ cells but not in ARPE-19 cells. (A) Schematic showing wild type c-Jun (wt-c-Jun) and non-phosphorylatable mutant c-Jun constructs. (B) Annexin V measurement of apoptosis following overexpression of wt-c-Jun or mut-c-Jun as indicated. (C) Immunoblots showing total and phosphorylated c-Jun following overexpression, SIRT1 loading control shown. (D) Levels of apoptosis following combined siRNA treatment in the presence or absence of non-target mut-c-Jun, (see materials and methods).

Co-silencing caspase 8 with JNK2 rescued cells from apoptosis (Fig. 4E). This effect was restricted to basal conditions since caspase 8 RNAi failed to rescue apoptosis following UV irradiation (data not shown). c-FLIP silencing alone induced apoptosis in HCT116 cells [29]. Co-silencing c-Jun, GSK3, or Fbw7 with c-FLIP failed to rescue cells from apoptosis (Fig. 6A), indicating that each of these pro-apoptotic intermediates function up-stream of c-FLIP (Fig. 7A-schematic). Thus basal and stress-induced apoptotic MAP kinase pathways converge at the level of c-FLIP. As expected, co-silencing caspase 8 with c-FLIP rescued cells from apoptosis (Fig. 6A and 6B).

### Over-expression of exogenous c-Jun induces apoptosis

We next asked if high level expression of c-Jun alone is sufficient to induce apoptosis. Over-expression of exogenous wild type c-Jun induced apoptosis in HCT116 cancer cells (Fig. 7A and 7B). Importantly, over-expression of a c-Jun phosphorylation mutant, in which S63, S73, T91 and T93 are all mutated to alanine, also induced apoptosis thus re-enforcing evidence for pro-apoptotic functions of hypo-phosphorylated c-Jun (Fig. 7A and 7B). Note that exogenous expression of another protein, SIRT1, does not induce apoptosis in HCT116 cells [22] indicating that the present results are specific to c-Jun. Protein expression from the c-Jun constructs and phosphorylation status shows phosphorylation at all sites examined on wild type c-Jun (Fig. 7C). This is indicative of cellular stress in response to exogenous gene over-expression. Note that this is in contrast to the sustained c-Jun hypo-phosphorylated status following RNAi-mediated gene silencing under the basal conditions employed throughout this work (see for example Fig. 3A). The c-Jun phosphorylation mutant lacked reactivity with anti-phospho-S63, S73 and T91 antibodies as expected (antibody to phosphoserine 93 was not available). Although both mutant and wild type exogenous c-Jun proteins were phosphorylated at T239 and S243 (Fig. 7C) we believe that this was non-essential for induction of apoptosis since endogenous c-Jun is required for apoptosis despite lack of C-terminal phosphorylation (see for example Fig. 4B-4D following JNK2 silencing). Significantly, over-expression of exogenous c-Jun did not induce apoptosis in non-cancer ARPE19 cells (Fig. 7B). These overall observations further re-enforce our conclusions (i) that elevated levels of hypo-phosphorylated c-Jun are pro-apoptotic in HCT116 cancer cells, and (ii) that the effect is specific for cancer cells (HCT116) and is not observed in non-cancer cells (ARPE19).

### Effects of c-Jun silencing are not attributable to off-target effects of c-Jun siRNA

The ability to rescue JNK2-depleted cells from apoptosis by combinatorial siRNA-induced gene silencing is strong evidence that our above results reflect specific gene silencing rather than general off-target effects of RNAi. This was confirmed for c-Jun by selective RNAi-targetting of endogenous wild type c-Jun in cells expressing an exogenous pro-apoptotic c-Jun mutant lacking a complementary mRNA recognition site for the c-Jun siRNA (Methods). For this purpose we used the c-Jun phosphorylation mutant already shown to be pro-apoptotic in HCT116 cells (see above; Fig. 7A and 7B). As before, JNK2 silencing alone induced apoptosis whilst co-silencing JNK2 with endogenous c-Jun rescued HCT116 cells from apoptosis (Fig. 7D). However, in those cells expressing the exogenous non-target c-Jun mRNA, wild-type c-Jun/JNK2 co-silencing failed to rescue cells from apoptosis (Fig. 7D). These results confirm that the observed effects of c-Jun silencing are attributable to selective gene silencing rather than to non-selective off-target effects of RNAi.

### Non-phosphorylated c-Jun accumulates in the insoluble nuclear fraction following JNK2 silencing

**Figure 8:**
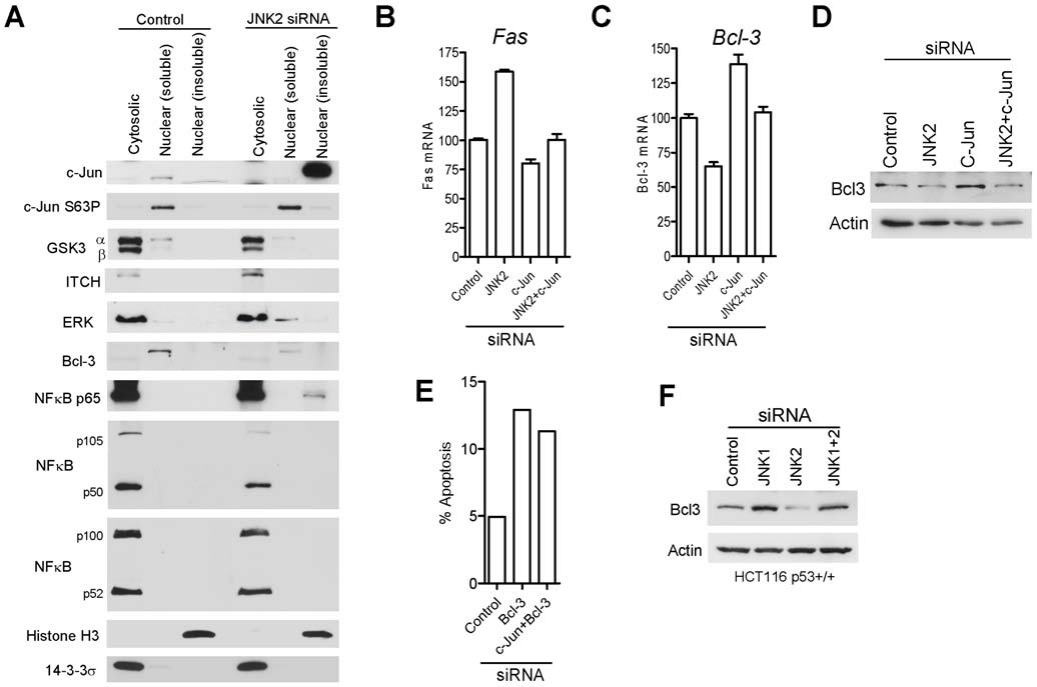
Non-phosphorylated c-Jun accumulates in the insoluble nuclear fraction following JNK2 silencing and down-regulates Bcl-3 expression in HCT116 p53+/+ cells. (A) Nuclear and cytoplasmic preparations following control and JNK2 siRNA treatment. Immunoblots show protein levels in the fractions indicated, equivalent cell number per lane. Histone H3 (nuclear insoluble) and 14-3-3σ (cytoplasmic) fractionation controls. (B and C) Quantitative PCR analysis of Fas and Bcl-3 mRNA. (D) Bcl-3 protein levels following siRNA treatment. (E) Levels of apoptosis following Bcl-3 or combined Bcl-3 and c-Jun siRNA treatment. (F) Immunoblots showing levels of Bcl-3 following siRNA treatment.

JNK2 silencing results in high level accumulation of non-phosphorylated c-Jun in HCT116 cancer cells (see above). Cell fractionation studies revealed that most, if not all, of the non-phosphorylated c-Jun protein accumulates in the nuclei of JNK2-silenced cells and selectively associates with the insoluble nuclear fraction (Fig. 8A). Prior to JNK2 silencing the c-Jun protein, present at low levels in HCT116 cells, predominated in the nucleoplasmic fraction (Fig. 8A). The accumulated hypo-phosphorylated c-Jun was physically separated from GSK3 and ERK, two kinases that can phosphorylate c-Jun (see Introduction), and also from ITCH, an E3 ligase involved in JNK1-mediated apoptosis: GSK3, ERK and ITCH all localised largely to the cytoplasmic fraction both before and after depletion of JNK2 (Fig. 8A). For ERK some additional protein was detectable in the soluble nuclear fraction following JNK2 silencing (Fig. 8A). The localisation of NFκB family members also showed little change following JNK2 silencing with the exception of p65 which showed some binding within the nuclear fraction (Fig. 8A). The insoluble nature of c-Jun's nuclear localisation following JNK2 silencing indicates that the accumulated hypo-phosphorylated c-Jun is complexed with sub-nuclear structures and/or chromatin. This is likely to include hypophosphoryated c-Jun bound at the c-Jun promoter (see above and Fig. 3). Non-cancer ARPE19 cells showed different sub-cellular localisations compared with HCT116 cancer cells in that c-Jun and Bcl-3 proteins were localised in the nuclear soluble and insoluble fractions under basal conditions (Fig. 8A and Fig. S5).

### JNK2 enhances, whilst c-Jun suppresses expression of Bcl-3, a pro-survival IκB protein

In an attempt to identify down-stream targets of JNK2/c-Jun we next screened for AP-1 targets reciprocally responsive to JNK2 and c-Jun silencing but with little or no response to combined JNK2 plus c-Jun co-silencing. We identified two AP-1 targets as fitting these criteria: namely FAS and Bcl-3 (Fig. 8B and 8C). It is well established that FAS is pro-apoptotic and, consistent with this, FAS expression was up-regulated upon JNK2 silencing, down-regulated upon c-Jun silencing, and these opposing effects were cancelled following JNK2 plus c-Jun co-silencing (Fig. 8B and 8C). These results indicate that up-regulation of FAS following JNK2 silencing is c-Jun-dependent.

In contrast Bcl-3 appeared to be down-regulated by c-Jun. Thus, c-Jun silencing resulted in increased Bcl-3 expression whilst JNK2 silencing correlated with Bcl-3 down-regulation (Fig. 8C and 8D). JNK1 silencing also increased Bcl-3 (Fig. 8F) consistent with its positive role in basal c-Jun functions. Bcl-3 down-regulation was clearly dependent upon c-Jun since co-silencing c-Jun with JNK2 negated their opposing effects on Bcl-3 expression (Fig. 8C and 8D). Thus JNK-2 silencing in HCT116 cancer cells causes down-regulation of Bcl-3 expression and this effect is mediated via c-Jun.

### Bcl-3 and c-Jun are pivotal in basal cancer cell survival

Since Bcl-3 has been implicated in abnormal cell survival we next asked if Bcl-3 is required for the survival of HCT116 cancer cells. RNAi-mediated silencing of Bcl-3 induced apoptosis in HCT116 cells (Fig. 8E). This effect could not be rescued by co-silencing c-Jun (Fig. 8E), indicating that the pro-survival function of Bcl-3 is down-stream of c-Jun. Our results suggest that the c-Jun pro-apoptotic mechanism operates, at least in part, via c-Jun-dependent down-regulation of Bcl-3. These observations are important and link the basal JNK2-mediated regulation of cancer cell survival with the NFκB pathway and identify c-Jun and Bcl-3 as reciprocal pivotal determinants of apoptosis versus survival respectively.

## Discussion

### Basal apoptosis versus stress-induced apoptosis

The programmed regulation of apoptosis under basal physiological conditions is crucial for normal mammalian development and, in the adult, for normal cellular turnover in individual tissue types. A further level of apoptotic control is induced in response to stress. Indeed, much of our understanding of the regulation of apoptosis in mammalian cells is based upon studies in which cells are analysed following exposure to defined stress conditions. From such studies it is becoming clear that different apoptotic programs are induced by different stimuli and are governed by cell type and physiological context [30].

In contrast to stress-induced apoptosis, the intrinsic regulation of apoptosis under basal conditions is poorly understood. To address this problem we have investigated cells in the absence of applied stress and explored the regulatory balance governing basal cell survival versus apoptosis. Such information is particularly important for understanding disease processes including cancer in which regulatory failure of basal apoptosis permits de-regulated growth and survival of cells in vivo.

### Basal cancer cell survival involves a novel apoptotic network

**Figure 9:**
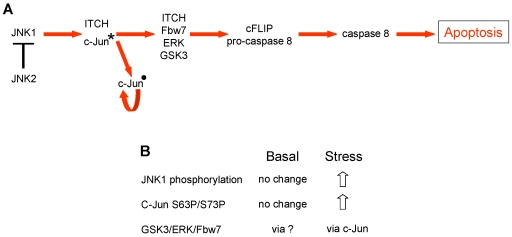
Basal survival of human cancer cells involves JNK2 suppression of a novel pro-apoptotic pathway. (A) JNK2 constitutively represses JNK1-mediated apoptosis. JNK1 is primed to initiate apoptosis which proceeds via the indicated intermediates (see Results). c-Jun dissociates from ITCH upon JNK2 depletion, loses S243P (*) and up-regulates its own transcription (•). (B) Distinctive characteristics of components of the MAP kinase pathway operating under basal conditions indicate that the regulation of apoptosis under basal conditions is mechanistically distinct from stress-induced apoptosis.

**Figure 10:**
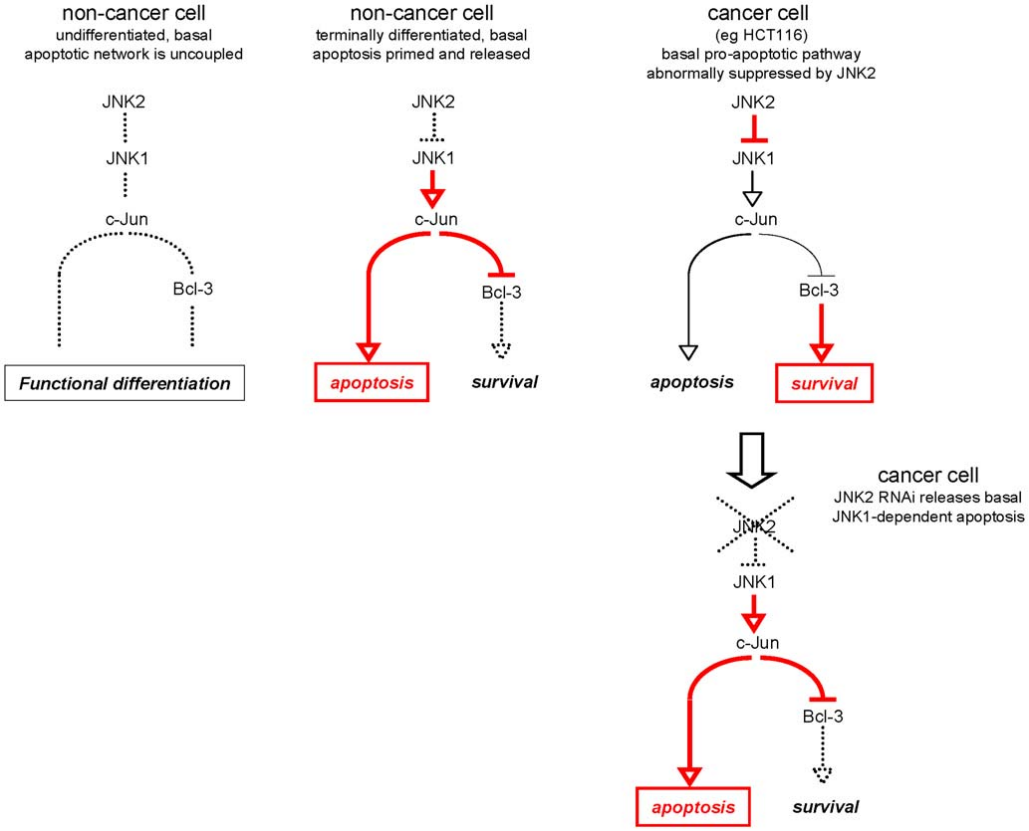
Basal regulation of apoptosis involves a novel network regulated by JNK2 and mediated via JNK1, c-Jun and Bcl-3. Hypothetical model for acquisition of an intrinsic apoptotic network in intestinal epithelial cells as they approach terminal differentiation. This network is subject to JNK2 which, abnormally, can enable cell survival and cancer via constitutive suppression of down-stream pro-apoptotic mediators under JNK1 control (see Discussion and Fig. 9).

Our results indicate that JNK2 is required for the survival of a range of human cancer cells cultured in the absence of applied stress. They also place JNK2 upstream of a novel regulatory network in which apoptosis (i) is driven by co-ordinated pro-apoptotic functions of JNK1 and c-Jun, (ii) is primed under basal conditions, (iii) is constitutively suppressed by JNK2, (iv) is linked with the NFκB survival pathway via Bcl-3/c-Jun, and (v) is mechanistically distinct from stress-induced apoptosis (Fig. 9 and Fig. 10). Moreover, the basal pro-apoptotic functions of JNK1 and c-Jun appear active despite the absence of site-specific phosphorylations known to be required for activation in response to stress. Following JNK2 depletion hypo-phosphorylated c-Jun protein translocates to the nucleus where it accumulates in the insoluble fraction and autoregulates its own expression. These changes in c-Jun are dependent upon hypo-phosphorylated JNK1 (Results and Fig. 9).

Downstream mediators of the basal pro-apoptotic pathway include components of the TNFα response pathway, namely ITCH, GSK3, ERK and Fbw7. However their functional interactions appear to be mechanistically distinct from those induced by stress (Results). This indicates that the regulation of basal cell survival/apoptosis is distinct from stress-induced apoptosis even though the same pro-apoptotic players are employed. It is possible that as yet uncharacterised protein-protein interactions govern basal apoptotic functions of hypo-phosphorylated JNK1, JNK2 and c-Jun. Such interactions may enable the formation of multi-protein complexes and basal apoptotic pathways distinct from those induced by stress.

Our results also indicate that the basal pro-apoptotic pathway is countered by the IκB protein Bcl-3. Once again JNK2 is the master regulator (Fig. 10). Mechanistically this is attributable to JNK2-mediated suppression of c-Jun, which we identify as down-regulator of Bcl-3 expression in HCT116 cancer cells. JNK2 depletion results in accumulation of c-Jun protein which, in turn, down-regulates Bcl-3 mRNA and protein levels (Results). Down-regulation of Bcl-3 directly impacts upon cell survival since we also demonstrate that RNAi-mediated depletion of Bcl-3 induces apoptosis despite the continued presence of JNK2 (Results).

The involvement of Bcl-3 in the basal regulation of cancer cell survival versus apoptosis is particularly interesting, especially since Bcl-3 is already identified as a putative oncoprotein [18], [31], [32], [33]. Bcl-3 has an atypical domain structure for an IκB protein. This confers distinct functional properties for Bcl-3. For example, in contrast to IκBα and IκBβ, the nuclear localisation signal on Bcl-3 lies outside the ankyrin repeat domain and enables transport of bound p50:p50 NFκB homodimers into the nucleus ([17] and references therein). Also, the seventh ankyrin repeat of Bcl-3, missing on IκBα and IκBβ, has been identified as a good candidate for interaction with the minor groove of DNA [17], possibly accounting for Bcl-3 complexing with DNA-bound NFκB homodimers.

The functional significance of Bcl-3 is unknown. Our discovery that this unusual IκB protein is reciprocally linked with JNK1/c-Jun in the basal regulation of apoptosis raises the possibility that Bcl-3 may function selectively under basal physiological conditions. Thus, during evolution, the functioning of Bcl-3 and IκBα/IκBβ may have diverged to regulate cell survival under basal versus stress-induced conditions respectively.

### Cancer versus non-cancer cells

Comparison of cancer and non-cancer cell lines indicates that the JNK2 requirement for cell survival is restricted to cancer cells (Results and Fig. 1). This is consistent with the reported viability of JNK2−/− mice (Introduction). For more in-depth comparison of cancer versus non-cancer cells we used HCT116 p53+/+ and ARPE19 cell lines. HCT116 p53+/+ are well characterised human epithelial colorectal cancer cells and have a stable karyotype. ARPE19 cells are partially differentiated retinal pigmented epithelial cells derived from non-cancerous tissue. ARPE19 have a normal karyotype and retain many of the structural and physiological properties of normal retinal pigmented epithelial cells *in vivo* [34], including polarised membrane expression of monocarboxylate transporters [35].

Our results demonstrate that HCT116 cancer cells are primed to undergo basal apoptosis in response to (i) JNK2 depletion, (ii) Bcl-3 depletion or (iii) exogenous over-expression of c-Jun. In contrast the ARPE19 non-cancer cells were refractory to each of these three conditions. These cancer-related differences may reflect differences in sub-cellular localisations of pro- and anti-apoptotic proteins such as c-Jun and Bcl-3 in cancer versus non-cancer cells (see Results, Fig. 8 and Fig. S5). Cancer-related differences in spatial proteomic fluxes may also influence the balance between pro- and anti-apoptotic effectors under basal conditions.

Another possibility is that altered metabolism and aerobic glycolysis, characteristic of cancer cells [36], also impact upon pro- and anti-apoptotic systems. In this context it is interesting to note that nucleolar JNK2 and SIRT1 proteins are both responsive to metabolic stress [37], [38] and both are also identified as cancer-related survival factors ([21], [22], [39], [40] and this present work). SIRT1 is an NAD-dependent de-acetylase and key regulator of aging, metabolism and homeostasis [37], [39]. SIRT1 protein turnover appears subject to JNK2 in human cell lines [22]. As yet the significance of this is unknown.

Cellular differentiation may also affect the balance of basal pro- and anti-apoptotic networks. For example, normal intestinal epithelial cells presumably become intrinsically primed to undergo basal apoptosis as a part of the normal process of cellular turnover. This process must be under stringent temporal control in order to prevent premature apoptosis on one hand, and to prevent delayed apoptosis and abnormal cellular accumulation on the other hand. On the basis of our current observations we suggest that components of the JNK and NFκB pathways may be exploited and co-ordinated to achieve this effect. We hypothesise that the basal JNK1/c-Jun pro-apoptotic pathway may be held in check by JNK2 until terminal apoptosis is required (represented schematically in Fig. 10). We further propose that during earlier stages of differentiation the JNK2/JNK1/c-Jun apoptotic network is non-operational. This would minimise the risk of premature apoptosis and also explain the observed resistance of undifferentiated non-cancer cells (Results) to JNK2 silencing or over-expression of c-Jun. However, at all stages of differentiation the cells are able to respond to stress since they express JNK and NFκB pathways which can be activated by stress-induced kinase signalling.

According to the above model for intrinsic apoptosis it is predicted that failure to engage the basal JNK1/c-Jun pro-apoptotic mechanism would compromise normal apoptotic turnover and so favour tumour formation. Indeed, this is exactly what is observed in JNK1-/- mice which exhibit pre-disposition to intestinal tumour formation [20]. Failure to alleviate JNK2-mediated suppression of JNK1/c-Jun would similarly favour tumour formation, and this is supported by our evidence for constitutive JNK2-mediated suppression of JNK1/c-Jun in a range of human colorectal cancer cell lines (this paper).

In summary, the link between JNK2/JNK1/c-Jun and Bcl-3 identifies pivotal roles for these proteins in the basal regulation of apoptosis versus cell survival. The discovery of a novel apoptotic network governing basal cell survival is crucial for understanding the intrinsic regulation of normal cellular turnover and opens new avenues for therapeutic intervention in human proliferative disease states.

## Materials and Methods

### Cell lines and transfection

Epithelial cancer cell lines used include human colorectal carcinoma HCT116 isogenic clones (p53^+/+^ and p53^−/−^; Bax^+/−^ and Bax^−/−^) [2], [41]; LoVo and RKO (wt p53), HT29 (mutant p53); breast carcinoma: MCF7 (wt p53) and osteosarcoma: U2OS (wt p53). Human non-cancer cell lines include ARPE-19 (retinal epithelial) and WI-38 (lung fibroblasts) and MCF7 10A (breast epithelial). MCF7 10A were grown in MEGM media supplemented with 100 ng/mL cholera toxin in the absence of FCS. Other cells lines were maintained and transfected using synthetic siRNAs (Dharmacon) as described previously [21], [22].

### siRNA sequences and mRNA quantification

Two independent siRNA target, previously validated for JNK1 or JNK2 [22] were used. siRNA's were typically used at a final concentration of 100 nM. Additional siRNA and primer sequences are provided in the Supplementary Information S1. Total RNA was isolated using RNeasy kit (Qiagen) following manufactures protocol and mRNA levels was measured by quantitative PCR using MJ DNA Engine Opticon with Qiagen SYBRgreen Quantitect kit. At least three determinations of mRNA levels were made.

### Immunoprecipitation and Immunoblotting

For immunoblotting whole cell lysates were prepared and processed in SDS buffer. Antibodies: anti-JNK1 (F-3), anti-c-Jun (sc-45), anti-c-Jun-p63 (KM-1), anti-p53 (DO-1), GSK3 (sc-7291) all Santa Cruz; JNK2, dual anti-phospho-SAPK/JNK (Thr^183^, Tyr^185^), anti-cleaved caspase 3, anti-caspase 7, anti-caspase 8, anti-c-Jun-p91, anti-c-Jun-p243, anti-FLIP, all Cell Signalling. Anti-c-Jun-p239 antibody (Abcam) was unable to detect c-Jun-239 phosphorylation under basal conditions or following JNK2 silencing. Bcl-3 (C-14) from Santa Cruz; NFκB p65 (3034) and NFκB p105/50 (3035) from Cell Signalling; ERK (9B3) and NFκB p100/52 from Abcam. c-Jun Ser63 phosphorylation antibody (KM-1, Santa Cruz). Anti-p21 (Pharmingen), anti-c-Jun-p73 (Upstate), and anti-ITCH (BD Transduction). Actin (AC40, Sigma) was used as an internal loading control throughout all experiments. Visualisation of bound antibodies was carried out using enhanced chemiluminescence kit (Roche).

For detection of c-Jun/ITCH protein complexes HCT116 p53+/+ cells were lysed in cold IP buffer (50 mM Tris pH 8, 150 mM NaCl, 1% NP40, 10 mM EDTA), in the presence of protease cocktail inhibitor (Roche), sonicated and centrifuged. The supernatant was percleared with protein G sephearse beads (Sigma) followed by assay of total protein (BCA kit, Pierce). 800 µg of total lysate was immunoprecipitated overnight at 4°C with 6 µg of c-Jun antibody (sc-45, Santa Cruz). Controls included c-Jun antibody with IP buffer only (no lysate), and IgG control. Immunocomplexes were captured with protein G sephearse, washed and eluted in denaturing SDS buffer. Eluates were immunoblotted for ITCH (BD Transduction) and c-Jun (Upstate).

### Chromatin Immunoprecipitation (ChIP)

ChIP was performed according to manufacturers protocol (EZ ChIP, Upsate). HCT116 p53^+/+^ cells were irradiated with 20 Jm^−2^ UV for 4 hours, or transfected with JNK2 siRNA for 48 hours. Additional details are provided in Supplementary Information S1.

### Stress treatment and apoptosis

Cells were treated with 20 Jm^−2^ UV for 4 h, or TNF-α (Calbiochem) at final concentration of 20 ng/mL at time points indicated. Apoptotic cells were identified by fluorescence activated cell sorting (FACS) using Annexin V-Fluos (Roche). Caspase 8, caspase 7 and caspase 3 were determined by immunoblotting.

### Nuclear and Cytoplasmic Extraction

Nuclear and cytoplasmic extraction was carried out using NE-PER kit (Pierce) according to manufactures instructions. Distribution of each protein is from a total of 750, 000 cells. The nuclear insoluble fraction employs a different buffer to the nuclear soluble fraction and accounting for slight changes in protein migration.

### Plasmids and overexpression studies

Flag tagged wild type human c-Jun and mutant c-Jun constructs have been described previously [10]. Mutant c-Jun contained (S63/S73 and T91/T93→ A substitutions). Transfection was carried out with 1 µg of empty vector (PC3.1, control) or c-Jun constructs as described previously [22] for 28 hours. Apoptosis was detected using annexin V labelling as described earlier. For combined overexpression and siRNA studies, cells were transfected with c-Jun (T91/93) siRNA as described earlier for 24 hours followed by transfection of empty control vector or mutant c-Jun construct (non recognisable to the siRNA, see Supplementary Information S1) for 24 hours.

## Supporting Information

Supplementary Information S1 Additional methods and supporting references
(0.05 MB DOC)

Figure S1 JNK2 constitutively suppresses JNK1 mediated apoptosis. Knock-down of JNK2 using a second independent siRNA causes apoptosis. This is rescued by a co-silencing with a second siRNA directed against JNK1. These results are consistent with the JNK1 and JNK2 siRNA used this study, (see also Materials and Methods).
(0.58 MB TIF)

Figure S2 JNK1 and JNK2 levels are unaffected by BCR-ABL or Lamin A/C siRNA treatment. Lamin A/C siRNA causes specific knock-down of Lamin A/C without affecting JNK1/JNK2 levels. BCR-ABL siRNA (BCR) has no target in the cell lines used in this investigation (negative control). The “stress sensor” p53 is not activated following transfection of an active (Lamin A/C) or inactive (BCR-ABL) siRNA.
(1.06 MB TIF)

Figure S3 JNK upstream kinases MKK4 and MKK7 are dispensable for JNK2 siRNA induced apoptosis in HCT116 p53+/+ cells. JNK2 siRNA mediated apoptosis was not rescued by co-silencing with MKK4 and was only partially rescued following combined MKK7 and JNK2 siRNA treatment.
(0.99 MB TIF)

Figure S4 Knock-down of JNK1 causes attenuation of c-Jun protein levels. The c-Jun blots in main figures have been under exposed to measure induction of c-Jun.
(0.99 MB TIF)

Figure S5 Apoptotic regulators fractionate into different compartments in ARPE-19 non-cancer cells. c-Jun and Bcl-3 localise in both the nuclear soluble and nuclear insoluble fractions under basal conditions compared to HCT116 p53+/+ cells where they are found exclusively in the nuclear soluble fraction, (see Figure 8A and Discussion).
(1.18 MB TIF)
