# Supplementary material for: Basal Cancer Cell Survival Involves JNK2 Suppression of a Novel JNK1/c-Jun/Bcl-3 Apoptotic Network
Source: PLoS One. 2009 Oct 6;4(10):e7305. doi: 10.1371/journal.pone.0007305 (PMC2752166; doi:10.1371/journal.pone.0007305)
Supplement: Supplementary Information S1 — Additional methods and supporting references (0.05 MB DOC) [file pone.0007305.s001.doc]

**Supplementary Information S1**

**Basal cancer cell survival involves JNK2 suppression**

**of a novel JNK1/c-Jun/Bcl-3** **apoptotic network**

Shafiq U Ahmed and Jo Milner

**Supplementary experimental section**

**siRNA transfections**

siRNA previously validated for ITCH [1], c-FLIP [2], Fbw7 [3], GSK3α/β [4], caspase 8 [5], c-Jun [6], MKK4 [7], MKK7 [8], Bcl-3 [9] and Fas [10] were used in this study. CKII was purchased from Qiagen (SI02660497). siRNA directed against c-Jun (T91/T93) region sense= 5’-CAUCACCACCACGCCGA(dTdT)-3’ and antisense 5’-GGUCGGCGUGGUGGUGAUG(dTdT)-3’ and ERK siRNA = sense 5’-GCAAUGACCAUAUCUGCUA(dTdT)-3’ and antisense 5’-UAGCAGAUAUGGUCAUUGC(dTdT)-3’.

**mRNA quantification**

Primers for SIRT1, JNK2, and internal controls GAPDH and Lamin A/C have been described [11,12]. Primers specific for JNK1 = (forward) 5’-CCAGGAAGGGACTATATTGATC-3’, (reverse) 5’-TCTCTCCTCCAAGTCCATAACT-3’, c-Jun (forward) 5’-ACGACCTTCTATGACGATGCCC-3’, (reverse) 5’-CCTCCTGCTCATCTGTCACG-3’; Bcl-3 (forward) 5’-TACAACAACCTACGGCAGACACC-3’, (reverse) 5’-ACGGTTTCTTGGCACTCGGTGTT-3’ and Fas (forward) 5’-CCAAGTGACTGACATCAACTC-3’, (reverse) 5’-CTCTTTGCATTGGTGTTGCTG-3’.

**Chromatin Immunoprecipitation (ChIP)**

2x10^7^ HCT116 p53+/+ cell lysate was crosslinked in 1% formaldehyde for 20 minutes, and sonicated to shear the DNA to an average of ~400bp. Samples were pre-cleared with protein G sepharose for 2 hours at 4^o^C. Protein-DNA complex in 1x10^6^ cells were captured overnight at 4^o^C with 5µg of antibodies against c- Jun (sc-45, Santa Cruz), p-Jun 63 (Upsate), p-Jun 73 (upstate) or corresponding control rabbit or mouse IgG (Upstate). Samples were washed and crosslinks removed by heating with proteinase K at 65^o^C overnight followed by purification of DNA, on columns provided. The IP captured DNA sample and sonicated genomic DNA extracts were subject to RT-PCR. The AP-1 sites on c-Jun were identified and numbered according to the accession (NM_002228). The two AP-1 sites on the *c-Jun* promoter (-1 and -120bp from start of +1 transcription site) was amplified using forward primer 5’- GAGGGTAGGAGAAAGAAGGGC-3’ and reverse primer 5’-GCTCAACACTTATCTGCTACCA-3’ resulting in a 217bp product (Figure 3B). ChIP enrichment with specific antibodies were normalised to signals from the non-expressed Goosecoid (GSC) control in the same sample and expressed as fold enrichment relative to IgG control [13].

**References**

1. Rossi M, De Laurenzi V, Munarriz E, Green DR, Liu YC, et al. (2005) The ubiquitin-protein ligase Itch regulates p73 stability. EMBO J 24: 836-848.

2. Longley DB, Wilson TR, McEwan M, Allen WL, McDermott U, et al. (2006) c-FLIP inhibits chemotherapy-induced colorectal cancer cell death. Oncogene 25: 838-848.

3. Welcker M, Orian A, Jin J, Grim JE, Harper JW, et al. (2004) The Fbw7 tumor suppressor regulates glycogen synthase kinase 3 phosphorylation-dependent c-Myc protein degradation. Proc Natl Acad Sci U S A 101: 9085-9090.

4. Grisouard J, Medunjanin S, Hermani A, Shukla A, Mayer D (2007) Glycogen synthase kinase-3 protects estrogen receptor alpha from proteasomal degradation and is required for full transcriptional activity of the receptor. Mol Endocrinol 21: 2427-2439.

5. Engels IH, Totzke G, Fischer U, Schulze-Osthoff K, Janicke RU (2005) Caspase-10 sensitizes breast carcinoma cells to TRAIL-induced but not tumor necrosis factor-induced apoptosis in a caspase-3-dependent manner. Mol Cell Biol 25: 2808-2818.

6. Lingor P, Koeberle P, Kugler S, Bahr M (2005) Down-regulation of apoptosis mediators by RNAi inhibits axotomy-induced retinal ganglion cell death in vivo. Brain 128: 550-558.

7. Kang YJ, Seit-Nebi A, Davis RJ, Han J (2006) Multiple activation mechanisms of p38alpha mitogen-activated protein kinase. J Biol Chem 281: 26225-26234.

8. Jaeschke A, Karasarides M, Ventura JJ, Ehrhardt A, Zhang C, et al. (2006) JNK2 is a positive regulator of the cJun transcription factor. Mol Cell 23: 899-911.

9. Westerheide SD, Mayo MW, Anest V, Hanson JL, Baldwin AS, Jr. (2001) The putative oncoprotein Bcl-3 induces cyclin D1 to stimulate G(1) transition. Mol Cell Biol 21: 8428-8436.

10. Saleem M, Kweon MH, Yun JM, Adhami VM, Khan N, et al. (2005) A novel dietary triterpene Lupeol induces fas-mediated apoptotic death of androgen-sensitive prostate cancer cells and inhibits tumor growth in a xenograft model. Cancer Res 65: 11203-11213.

11. Ford J, Jiang M, Milner J (2005) Cancer-specific functions of SIRT1 enable human epithelial cancer cell growth and survival. Cancer Res 65: 10457-10463.

12. Jiang M, Milner J (2003) Bcl-2 constitutively suppresses p53-dependent apoptosis in colorectal cancer cells. Genes Dev 17: 832-837.

13. Baxter EW, Cummings WJ, Fournier RE (2005) Formation of a large, complex domain of histone hyperacetylation at human 14q32.1 requires the serpin locus control region. Nucleic Acids Res 33: 3313-3322.
