# Supplementary figures and images for: Basal Cancer Cell Survival Involves JNK2 Suppression of a Novel JNK1/c-Jun/Bcl-3 Apoptotic Network
Source: PLoS One. 2009 Oct 6;4(10):e7305. doi: 10.1371/journal.pone.0007305 (PMC2752166; doi:10.1371/journal.pone.0007305)

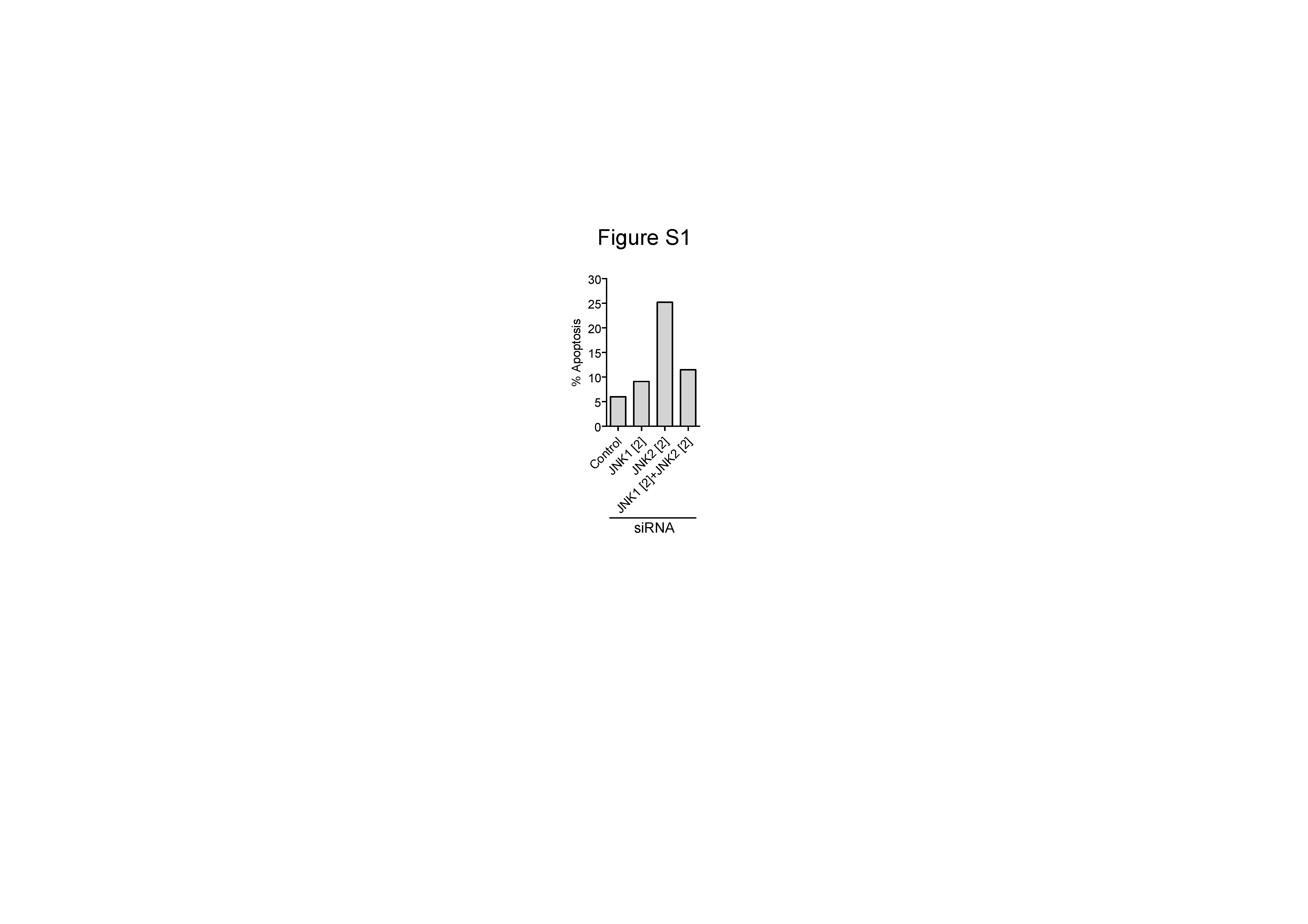

Supplement: Figure S1 — JNK2 constitutively suppresses JNK1 mediated apoptosis. Knock-down of JNK2 using a second independent siRNA causes apoptosis. This is rescued by a co-silencing with a second siRNA directed against JNK1. These results are consistent with the JNK1 and JNK2 siRNA used this study, (see also Materials and Methods). (0.58 MB TIF) [file pone.0007305.s002.tif]

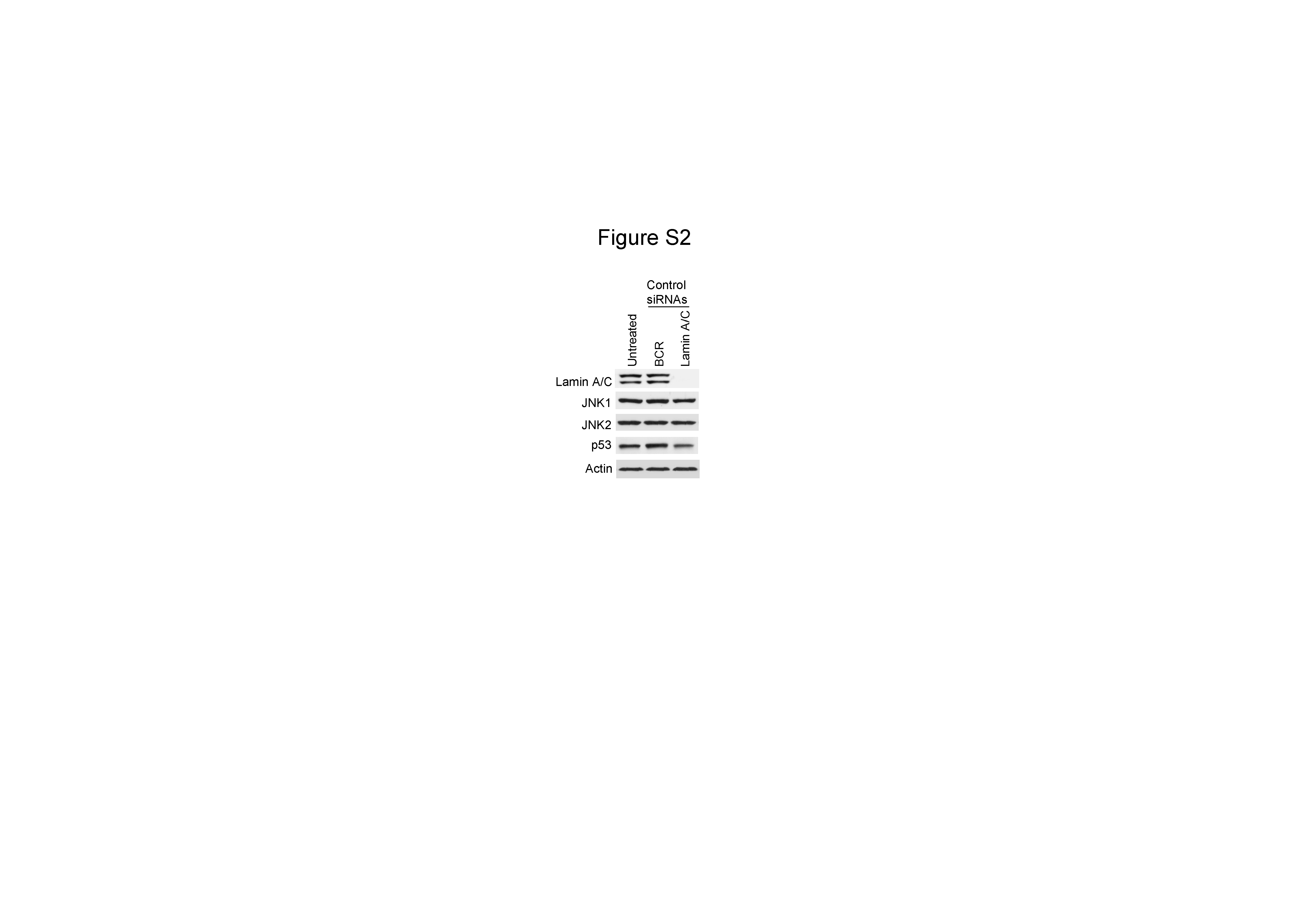

Supplement: Figure S2 — JNK1 and JNK2 levels are unaffected by BCR-ABL or Lamin A/C siRNA treatment. Lamin A/C siRNA causes specific knock-down of Lamin A/C without affecting JNK1/JNK2 levels. BCR-ABL siRNA (BCR) has no target in the cell lines used in this investigation (negative control). The “stress sensor” p53 is not activated following transfection of an active (Lamin A/C) or inactive (BCR-ABL) siRNA. (1.06 MB TIF) [file pone.0007305.s003.tif]

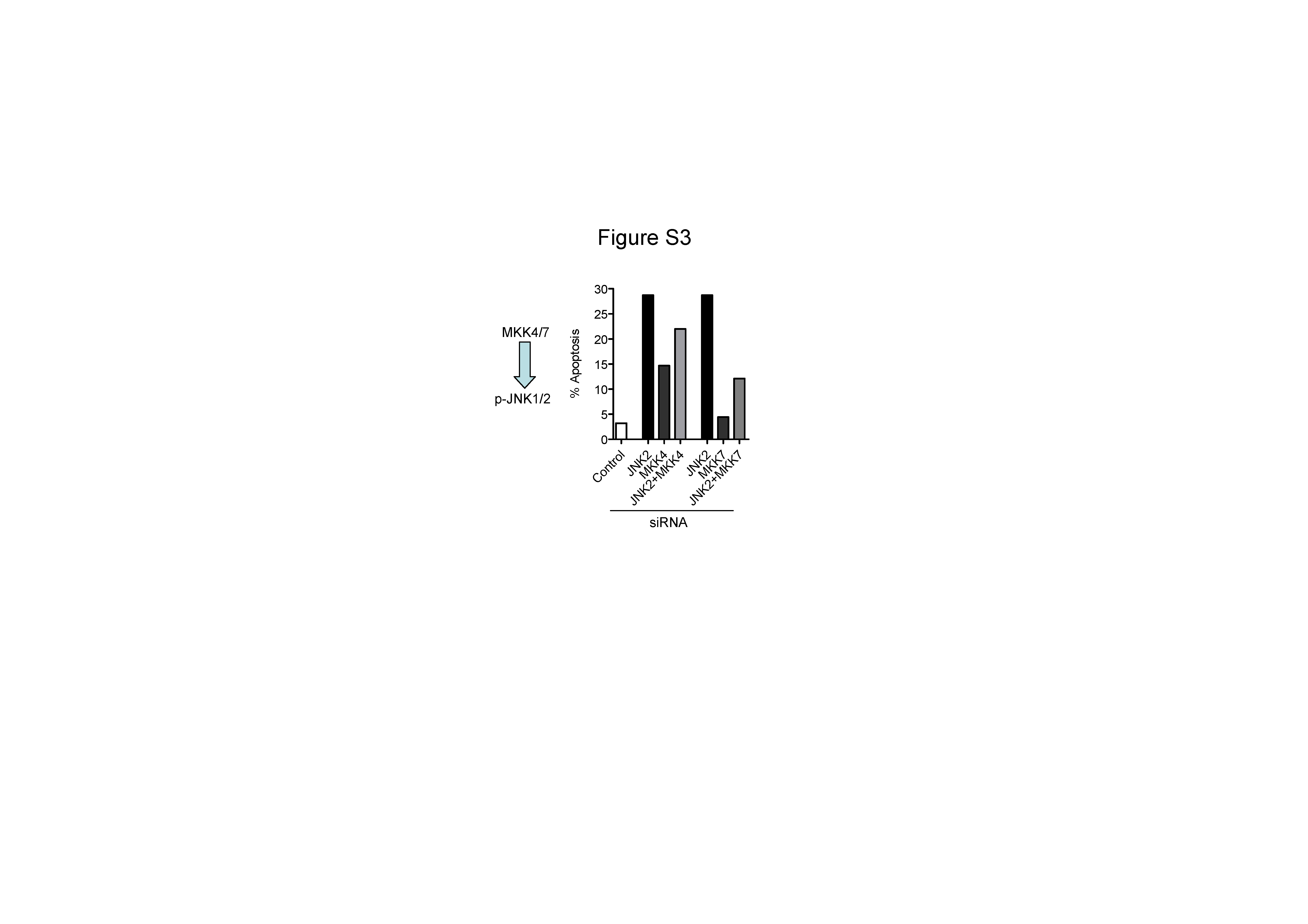

Supplement: Figure S3 — JNK upstream kinases MKK4 and MKK7 are dispensable for JNK2 siRNA induced apoptosis in HCT116 p53+/+ cells. JNK2 siRNA mediated apoptosis was not rescued by co-silencing with MKK4 and was only partially rescued following combined MKK7 and JNK2 siRNA treatment. (0.99 MB TIF) [file pone.0007305.s004.tif]

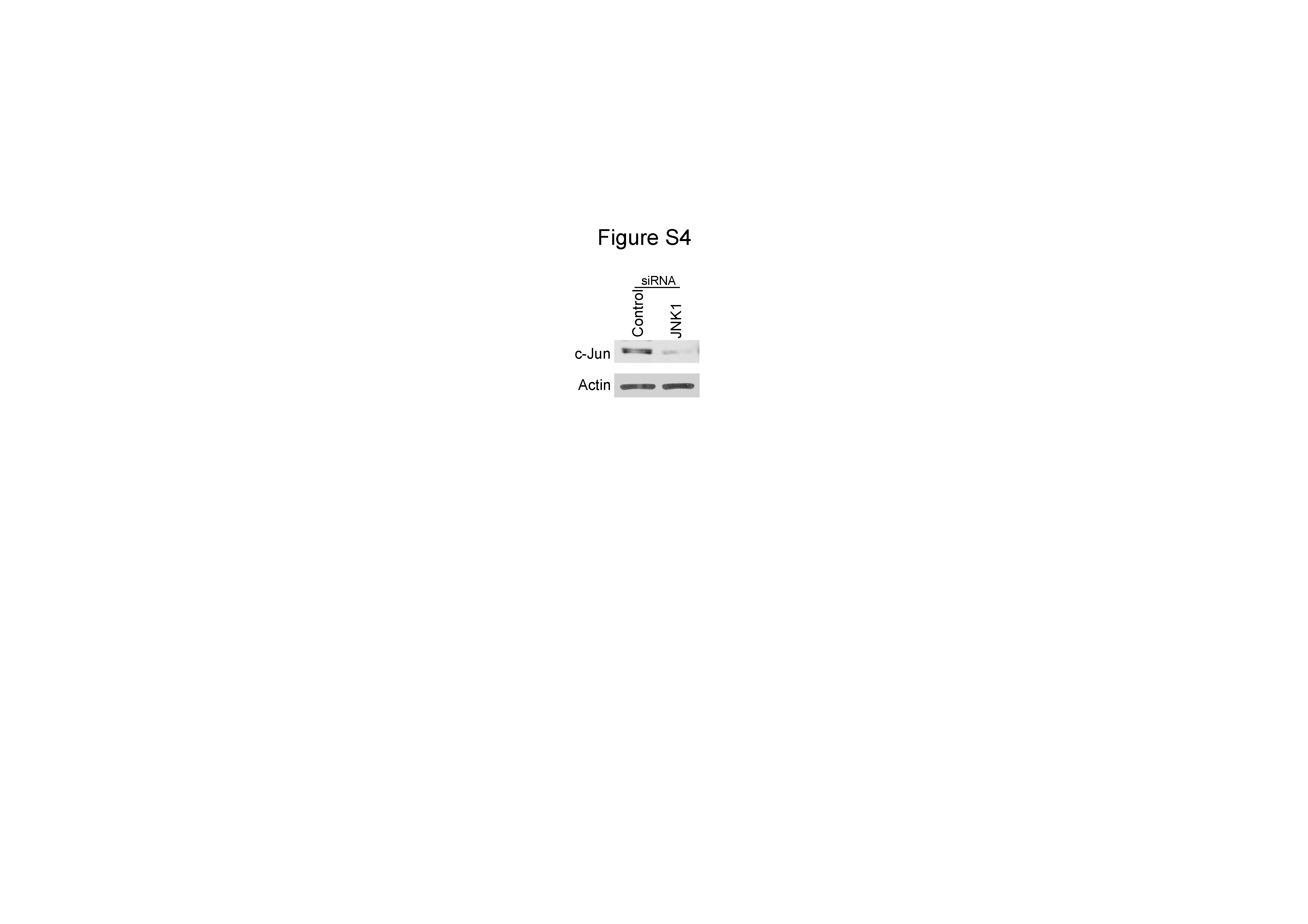

Supplement: Figure S4 — Knock-down of JNK1 causes attenuation of c-Jun protein levels. The c-Jun blots in main figures have been under exposed to measure induction of c-Jun. (0.99 MB TIF) [file pone.0007305.s005.tif]

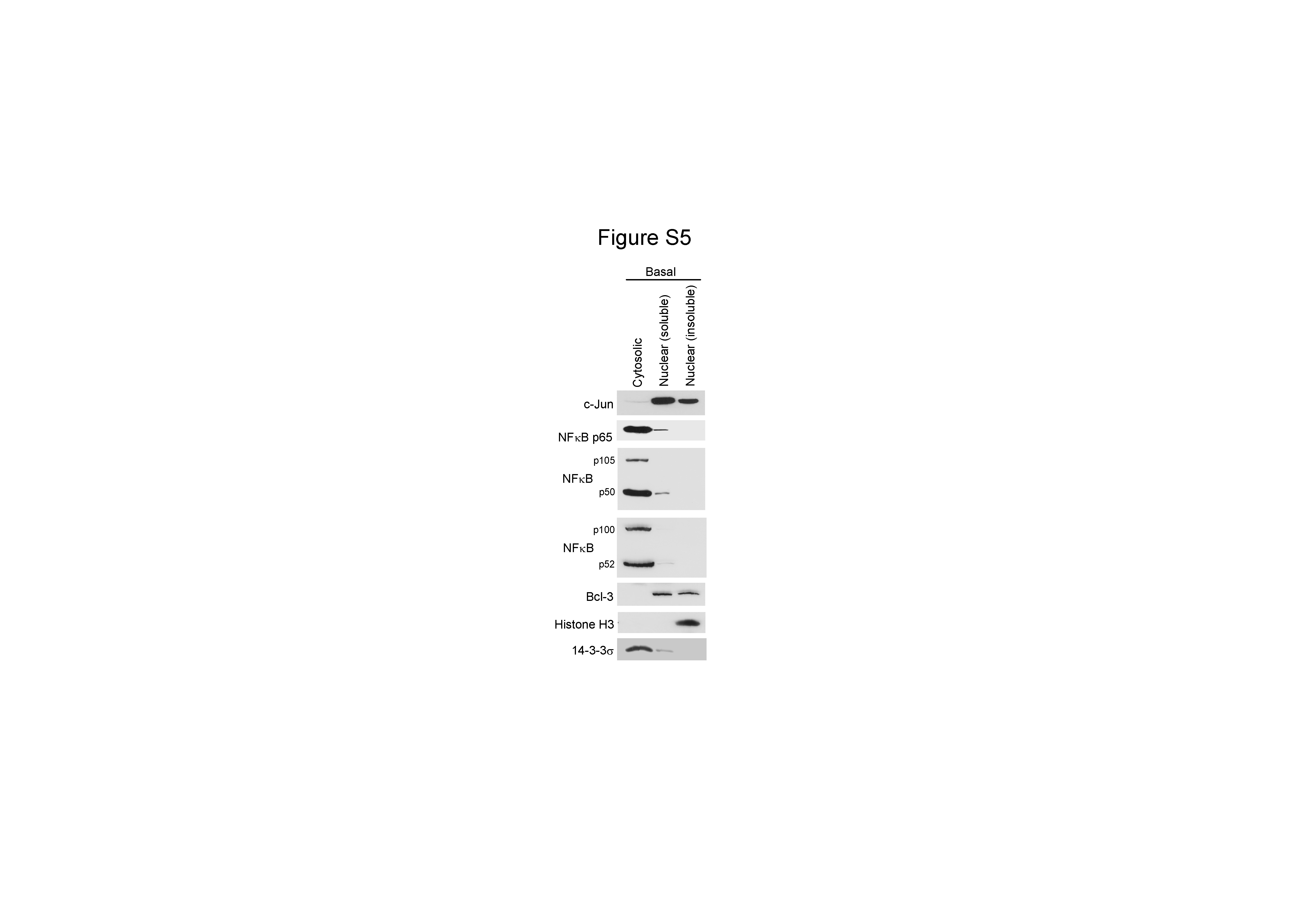

Supplement: Figure S5 — Apoptotic regulators fractionate into different compartments in ARPE-19 non-cancer cells. c-Jun and Bcl-3 localise in both the nuclear soluble and nuclear insoluble fractions under basal conditions compared to HCT116 p53+/+ cells where they are found exclusively in the nuclear soluble fraction, (see Figure 8A and Discussion). (1.18 MB TIF) [file pone.0007305.s006.tif]
